# Testing a Photo Story Intervention in Paper Versus Electronic Tablet Format Compared to a Traditional Brochure Among Older Adults in Germany: Randomized Controlled Trial

**DOI:** 10.2196/12145

**Published:** 2018-12-06

**Authors:** Shu Ling Tan, Amanda Whittal, Sonia Lippke

**Affiliations:** 1 Institute of Sport and Exercise Sciences Department of Social Sciences of Sports University of Münster Münster Germany; 2 Health Psychology and Behavioral Medicine Department of Psychology and Methods Jacobs University Bremen Bremen Germany; 3 Bremen International Graduate School of Social Sciences Jacobs University Bremen Bremen Germany

**Keywords:** photo story, traditional brochure, health literacy, communication, older adults, tablet intervention, electronic/information technology, primary care consultation

## Abstract

**Background:**

To increase effective communication in primary care consultations among older adults in Germany, the photo story is considered to be a useful tool based on Bandura’s social cognitive theory. With information technology helping to increase effective communication, the use of tablets is gaining attention in health care settings, especially with older adults. However, the effectiveness of tablet technology and photo stories has rarely been tested.

**Objective:**

The aim is to compare the effectiveness of a photo story intervention to a traditional brochure. Both were delivered either in paper or tablet format.

**Methods:**

A trial was conducted with 126 older adults, aged 50 years and older, who were approached and recruited by researchers and administrative staff from senior day care, doctors in rehabilitation centers, and trainers in sports clubs in Germany. Open and face-to-face assessment methodologies were used. Participants were randomly assigned to one of four intervention conditions: traditional brochure in paper format (condition 1) and tablet format (condition 2), and photo story in paper format (condition 3) and tablet format (condition 4). Each participant received a questionnaire and either the traditional brochure or photo story in a paper or tablet version. To evaluate the effectiveness of each intervention, participants completed evaluation questionnaires before and after each intervention. The second part of the questionnaire measured different indicators of health literacy, communication skills, health measurements, and possible underlying mechanisms.

**Results:**

Compared to the traditional brochure, participants considered the photo story easier to understand (*t*_124_=2.62, *P*=.01) and more informative (*t*_124_=–2.17, *P*=.03). Participants preferred the paper format because they found it less monotonous (*t*_124_=–3.05, *P*=.003), less boring (*t*_124_=–2.65, *P*=.009), and not too long (*t*_124_=–2.26, *P*=.03) compared to the tablet format. Among all conditions, the traditional brochure with a tablet (condition 2) was also perceived as more monotonous (mean 3.07, SD 1.08), boring (mean 2.77, SD 1.19), and too long to read (mean 2.50, SD 1.33) in comparison to the traditional brochure in paper format (condition 1). Moreover, the participants scored significantly higher on self-referencing on the traditional brochure in paper format (condition 1) than tablet format for both types of the brochure (conditions 2 and 4).

**Conclusions:**

Traditional brochures on a tablet seem to be the least effective communication option in primary care consultations among all conditions for older adults. The findings might be specific for the current generation of older adults in Germany and need to be replicated in other countries with larger sample sizes. Although information technology brings advantages, such as effective interventions in different fields and settings, it may also come with several disadvantages, such as technical requirements of the users and devices. These should be considered when integrating information technology into wider situations and populations.

**Trial Registration:**

ClinicalTrials.gov NCT02502292; https://clinicaltrials.gov/ct2/show/NCT02502292 (Archived by Webcite at http://www.webcitation.org/747jdJ8pU)

## Introduction

The involvement of older people in medical decision making and the barriers they experience during conversations with health care professionals have been a prominent focus in clinical settings [1]. One study revealed that the most common barriers include difficulties understanding medical information [1]. Almost half of the patients preferred to play a passive role in medical decision making, but still might want more information about their health care, and 27.5% of patients were not asked for their opinion at all. Therefore, this study concluded that information and communication are basic requirements for everyone to participate in medical decision making. According to Clayman and colleagues [2], the ability to efficiently communicate with health care providers is an important element of proper self-care. This is especially important for individuals with limited health literacy: to be able to obtain, understand, and recall information from their doctors. Therefore, this study aims to develop relevant and beneficial interventions, which address the aforementioned aspects including useful information and communication, to assist older adults with low health literacy during their care consultations and increase their ability to comprehend health-related information.

Narrative communication as a tool has been strongly recommended in previous studies [3], as it is an effective approach to interaction regardless of literacy level. This assumption is based on Bandura’s social cognitive theory [4] because a photo story with a comic layout, modeled pictures, and bubble conversations addresses the three main sources of self-efficacy and outcome expectations. In his self-efficacy theory, Bandura hypothesized that self-efficacy influences choice of activities, goal setting, and initiation of behavior, as well as coping efforts after commencement of the behavior (maintenance). Self-efficacy controls how much effort one invests and how persistent one is in investing more effort to deal with obstacles and adverse experiences. In addition, performance feeds back to self-efficacy expectation creating a reciprocal effect. Self-efficacy is linked with goals in that the higher self-efficacy is, the more likely people are to set a goal. Self-efficacy also affects outcome expectations: individuals with higher self-efficacy are more likely to perceive outcomes as more favorable [4,5].

Bandura’s self-efficacy theory describes four different influence procedures or sources of self-efficacy: performance accomplishments, vicarious experience, verbal persuasion, and emotional arousal. They can all directly influence self-efficacy and thereby have a mediated effect on behavior [4,5]. Thus, they represent targets of interventions to create or alter self-efficacy and enable people to perform a behavior to attain a set goal.

Personal experiences or performance accomplishments are often called *mastery experiences*. Personal experiences have been found to have the highest impact on self-efficacy beliefs and thereby on future behavior. However, an avatar would only provide this experience when using information technology, but not personal contact. Thus, of higher importance are the other sources. Vicarious experience is the second source and includes all experiences observed by the individual, like in a photo story or in a traditional brochure on a tablet. Model learning builds on vicarious experiences by observing others and drawing conclusions for one’s own behavior and its predictors. The more similar the model (ie, the observed other person) is to the individual, the more likely it is that the observations have an impact on the individual [4,5].

The third and weaker factor, compared to the first two sources, is verbal persuasion. Verbal feedback and instruction can come from other people, texts, or self-instruction [4,5]. Such feedback is not usually possible with photo stories or in a traditional brochure on a tablet. However, the last and least strong source is a physiological state of emotional arousal; such arousal can be elicited by material such as a photo story or in a traditional brochure on a tablet.

Photo Story is an alternative tool in a comic layout with modeled pictures and bubble conversation [6], which is considered to be one of the most useful health literacy strategies to engage people in effective communication through the process of reflection and critical thinking [7]. This tool has been used in several contexts; for example, to promote healthy eating in a Latino community [8], to support older adults with limited health literacy during doctor-patient communication [9], and to improve depression literacy and help-seeking behaviors [6]. Moreover, a previous study highlighted the value of comic strips, which share similar characteristics with photo stories, as a format for health information [10]. Despite this, there is a lack of systematic studies that examine the effectiveness of photo story interventions, especially in older adults. This study aims at filling this gap.

The usefulness of information technology electronic devices, such as tablets, in the context of primary care consultations for older adults is not yet well understood [11]. A systematic review and meta-analysis suggested older adults have the potential to benefit from the use of tablet technology, especially in health care settings [12]. Information technology can be used in different ways to promote health literacy among people with low literacy. A study by Wang and colleagues [13] tested the effectiveness of a story-based video as an educational tool to increase people’s comprehension of prostate health terminology. Their findings showed that comprehension significantly increased for 13 of 32 terms. The researchers concluded that story-based education by means of videos has the potential to increase comprehension and support shared decision making [13].

Another previous study tested the readability of discharge instructions for hospital patients, either by means of electronic templates for specific diagnoses or by doctors for whom no templates were made available. Results showed that the readability of electronic diagnosis-specific templates was better than the instructions generated by the doctors [14]. Yet another study compared tablet and paper formats of a tablet-based consent process for a mock clinical trial among older adults. The results showed that the older adults accepted the tablet-based consent process and it was feasible to implement, although they took a longer time to complete the tablet format compared to the paper format [15].

Despite the wide range of studies available, there is a visible lack of research, especially randomized controlled trials with study interventions, to test the effectiveness of the photo story for increasing health-related understanding and the usefulness of electronic devices, such as tablets [12]. A systematic review assessing the evidence for the effectiveness of such interventions concluded that there is a lack of consistent evidence for effective interventions [16].

Based on the presented evidence, it seems that older adults with low health literacy can gain from innovative solutions that help to improve understanding and communication in health settings. Such innovative solutions can make use of the advances in technology occurring today but require interventions to test effectiveness and find the right solution for the target population. This study therefore provides insight into a photo story intervention as a potentially effective communication tool. The following research questions were tested:

1. Which intervention do older adults appreciate more: the photo story or the traditional brochure?
2. Which format do older adults prefer in the intervention: the paper format or the tablet format?
3. Which condition of the interventions (the photo story or the traditional brochure provided on paper or on a tablet) is more effective in increasing older adults’ communicational self-efficacy and behavioral intentions in the context of primary care consultations in Germany, and has effects on different aspects of health literacy (transportation, identification, self-referencing)?

## Methods

### Participants

Participants were approached and recruited by researchers via administrative staff of senior day care, rehabilitation centers, and sports clubs in Germany. Data collection took place in 2015. Only participants who met the following inclusion criteria were approached to participate in the study: (1) aged 50 years or older, (2) no cognitive impairments with average literacy enabling them to complete a questionnaire without help, and (3) German language proficiency.

### Procedure

To guarantee a standardized approach, all researchers completed training before the start of the data collection process. Following training, each researcher was assigned to a sports club or rehabilitation center, and then they contacted the facility to arrange appointments with the participants to collect the data. The researchers introduced themselves to participants and provided information sheets, which contained both the aim and basic information about the study.

Ethical approval was applied for and received from the Deutsche Gesellschaft für Psychologie (German Association for Psychology). It was conducted in line with the American Psychological Association’s ethical principles and the 1964 Helsinki Declaration and its later amendments or comparable ethical standards. The study was registered at ClinicalTrials.gov (NCT02502292), where the description of this study with its objectives, designs, methodologies, and interventions were submitted with several purposes: to decrease publication and outcome reporting biases, and to promote the implementation of ethical obligations to participants [17].

After signing the consent form, each participant received a questionnaire and either the traditional brochure or photo story in a paper or tablet version. The researcher was present to answer participants’ questions or concerns. The intervention was developed by partners of the Intervention Research on Health Literacy among the Ageing Population consortium to improve older adults’ health literacy and communication skills during care consultations. The photo story and traditional brochure used in this study were developed based on both previous literature [3,6,8] and the outcomes of focus group discussions conducted in the Netherlands and Hungary [9,16]. The traditional brochure contained only text health information, whereas the photo story contained storylines and sketches with photographs in a clinic setting between a doctor and a patient, with added speech and thought bubbles according to the scripts. More details have been published elsewhere [9,16], but are distinct from this study. Participants in the two tablet conditions were shown the traditional brochure and the photo story in the form of a PDF (Portable Document Format). After they returned their brochures, the second part of the questionnaire was given to the participants to complete. At the end of the study, all participants received a debriefing statement with the researchers’ contact details in case they had questions or suggestions regarding the study.

### Measures

The questionnaire consisted of two parts: the first part contained questions regarding sociodemographics, perceived health and well-being measurements, morbidities, and the frequency of doctor consultations. The health literacy Set of Brief Screening Questions was also utilized, with three 5-point Likert scale questions (eg, “How often do you need help to read the information papers from the hospital?” [18]). In addition, questions regarding the general level of communicative self-efficacy (Ask, Understand, Remember Assessment; AURA) were included, such as “Is it easy for you to ask your doctor questions?”; Cronbach alpha was .83 [2].

In the second part, questions related to each story of the traditional brochure, including domain-specific self-efficacies (eg, “You have the feeling that your doctor might not give you enough attention, is it easy for you to make him aware of it?” rated from 1=no, not at all to 5=yes, absolutely) and behavioral intentions items (eg, “You have the feeling that your doctor doesn’t give you enough attention, would you make him aware of this feeling?”) [19-21]. The questions regarding self-efficacy included aspects of attention calling, social support mobility, clarifications, medication help seeking, instruction checking, question asking, and question developing. Cronbach alpha was determined to be .70 for self-efficacy and .77 for behavioral intention. To investigate the underlying mechanisms of effective communication, questions about self-referencing [22], identification, and transportation [23] were also included. This was followed by evaluation questions regarding the traditional brochure and photo story, such as “Did you find the booklet hard to understand?”

### Analyses

**Figure 1:**
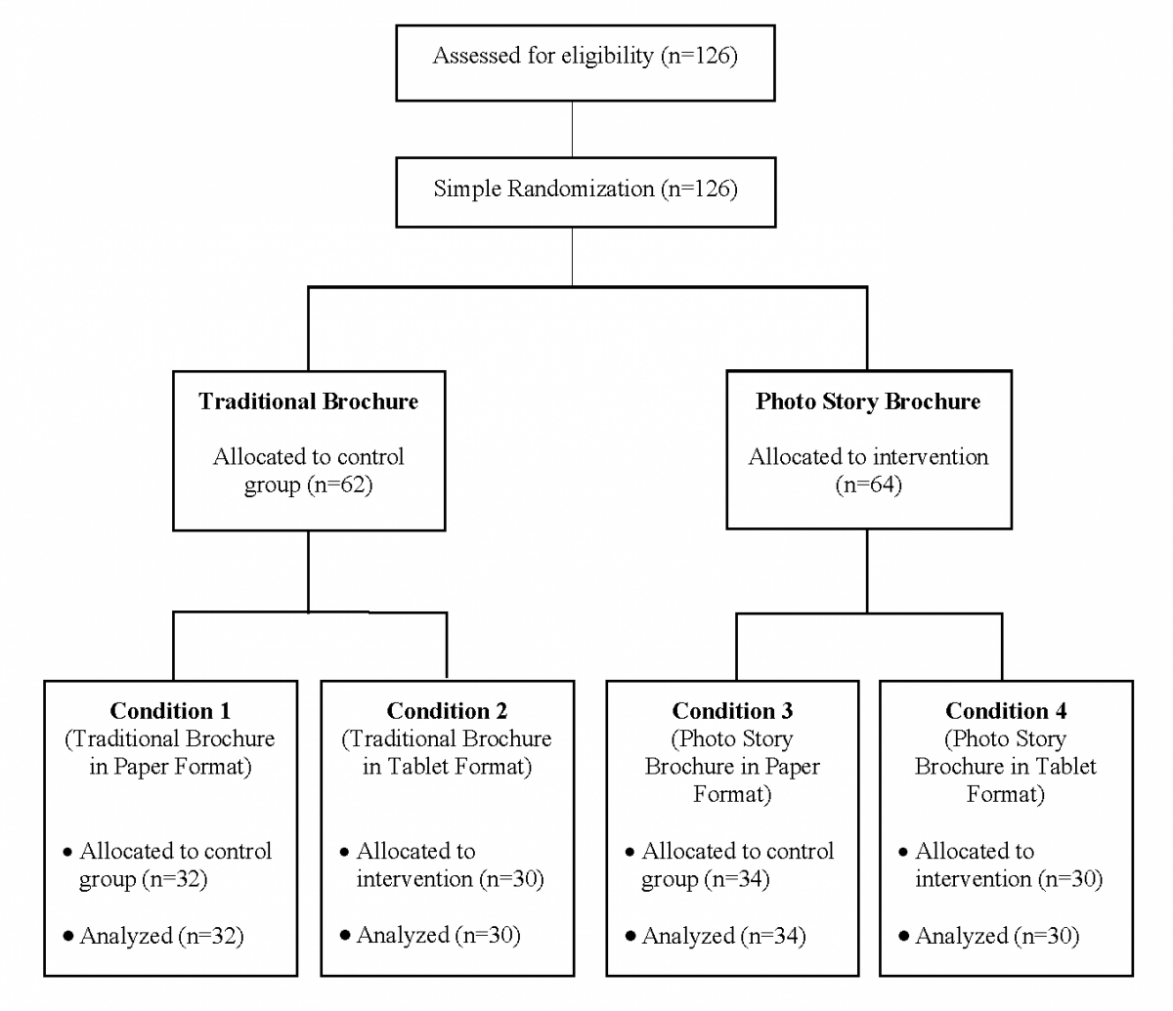
Consolidated Standards of Reporting Trials (CONSORT) flow chart.

Statistical analyses were carried out with SPSS version 25. As shown in Figure 1, simple randomization checks were performed between participants of the control groups (traditional brochure and paper format) and the interventions (photo story and tablet format). We used multivariate analyses of variance to (1) to investigate perceived health, frequency of doctor visits, and communication self-efficacy across the four conditions; (2) to gain insight into the evaluations of the effectiveness of the interventions across all four conditions; and (3) to examine the intervention’s effectiveness in increasing older adults’ communication self-efficacy and behavioral intentions, as well as the underlying mechanisms (ie, self-referencing, identification, and transportation) across all four conditions. These analyses were followed by polynomial contrasts and post hoc multiple comparisons. In addition, we conducted two independent *t* tests with seven evaluation variables to compare the means of the two independent groups: photo story and traditional brochure, as well as the mean differences between paper and tablet formats.

## Results

A total of 126 participants, aged 50 years and older (mean 71.85, SD 10.13 years), were recruited and randomly assigned with simple randomization to one of four test conditions. In total, 61.9% (78/126) of the participants were female and 38.1% (48/126) were male. Further, 45.2% (57/126) of the participants had no chronic disease, 35.7% (45/126) had one chronic disease, and 17.5% (22/126) had more than one chronic disease, including cardiovascular disease, respiratory disease, diabetes, rheumatism, or diseases of the musculoskeletal system. In total, 67.5% (85/126) were married and/or in a long-term relationship, and 78.6% (99/126) were retired.

### Health Measurements

Both means and standard deviations for perceived health, frequency of doctor visits, and communication self-efficacy (AURA) across the four conditions are displayed in Table 1.

As can be seen in Table 1, the participants perceived their health average to be between “satisfactory” and “well” and, on average, participants consulted their doctors between every 3 and 6 months. Regarding their communication self-efficacy, during their doctor visits, participants perceived themselves to be able to understand their doctors’ instructions. However, there was no statistically significant difference in perceived health, frequency of doctor visits, and communication self-efficacy between the four different conditions (*F*_9,292_=0.597, *P*=.79, Wilk λ=0.96, partial η^2^=.015). There was also no significant effect among different groups for perceived health (*F*_3,122_=0.95, *P*=.42, partial η^2^=.023), frequency of doctor visits (*F*_3,122_=1.20, *P*=.31, partial η^2^=.029), and communication self-efficacy (*F*_3,122_=0.20, *P*=.89, partial η^2^=.005).

### Photo Story Versus Traditional Brochure

Three significant differences between the two groups of the photo story and traditional brochure conditions were found indicating that participants considered the photo story to be easier to understand (*t*_124_=2.62, *P*=.01) and more informative (*t*_124_=–2.17, *P*=.03) in comparison to the traditional brochure (see Table 2).

### Paper Versus Tablet Format

Comparing the paper format (n=66) with the tablet format (n=60) across the same seven variables, three significant differences between these two groups were seen: participants preferred the paper format because they found it less monotonous (*t*_124_=–3.05, *P*=.003), less boring (*t*_124_=–2.65, *P*=.009), and not too long (*t*_124_=–2.26, *P*=.03) compared to the tablet format (see Table 3).

**Table 1:** Means and standard deviations of perceived health, frequency of visit to doctor and communicative self-efficacy, across four conditions.

Types of brochures	Traditional, mean (SD)		Photo story, mean (SD)		Total (N=126)	*P* value
	Paper (n=32)	Tablet (n=30)	Paper (n=34)	Tablet (n=30)		
Perceived health	3.28 (0.96)	3.60 (0.93)	3.35 (0.85)	3.57 (0.86)	3.44 (0.90)	.42
Frequency visit doctor	4.34 (0.97)	4.60 (1.13)	4.38 (0.99)	4.77 (0.94)	4.52 (1.01)	.31
Communicative self-efficacy (AURA^a^)	4.06 (0.73)	4.06 (0.67)	3.94 (0.91)	4.08 (0.79)	4.03 (0.77)	.89

^a^AURA: Ask, Understand, Remember Assessment.

**Table 2:** Means and standard deviations of the evaluations between the traditional brochure (conditions 1 and 2) and photo story (conditions 3 and 4) tool (N=126).

“Did you find the booklet...”^a^	Traditional brochure (n=62), mean (SD)	Photo story brochure (n=64), mean (SD)	*P* value
Hard to understand?	2.03 (1.25)	1.52 (0.94)	.01
Interesting?	3.56 (1.22)	3.63 (1.23)	.78
Monotonous?	2.66 (1.23)	2.48 (1.16)	.41
Boring?	2.34 (1.21)	2.28 (1.20)	.79
Enjoyable?	2.42 (1.22)	2.56 (1.15)	.50
Informative?	3.37 (1.39)	3.86 (1.13)	.03
Too long?	2.13 (1.34)	2.23 (1.24)	.65

^a^Answers were given on a five-point rating scale with 1=no, not at all; 2=rather not; 3=neither; 4=yes to some extent; 5=yes, completely.

**Table 3:** Means and standard deviations of the evaluations between paper (conditions 1 and 3) and tablet (conditions 2 and 4) formats (N=126).

“Did you find the booklet...”^a^	Paper format (n=66), mean (SD)	Tablet format (n=60), mean (SD)	*P* value
Hard to understand?	1.77 (1.12)	1.77 (1.16)	.98
Interesting?	3.68 (1.33)	3.50 (1.10)	.41
Monotonous?	2.27 (1.18)	2.90 (1.12)	.003
Boring?	2.05 (1.18)	2.60 (1.17)	.009
Enjoyable?	2.56 (1.31)	2.42 (1.03)	.49
Informative?	3.77 (1.35)	3.45 (1.20)	.16
Too long?	1.94 (1.24)	2.45 (1.29)	.03

^a^Answers were given on a five-point rating scale with 1=no, not at all, 2=rather not, 3=neither; 4=yes to some extent, 5=yes, completely.

### Traditional Brochure Versus Photo Story in Paper and Tablet Formats

There were no statistically significant group differences among the conditions in the evaluation variables (*F*_21,334_=1.34, *P=*.15, Wilk’s λ=0.79, partial η^2^=.075). Nonetheless, the results showed significant differences in the variables of the evaluation item that assessed the understanding of the condition of brochure and format (*F*_3,122_=2.91, *P*=.04, partial η^2^=.067), the item that assessed whether the condition of brochure and format was monotonous (*F*_3,122_=3.51, *P*=.02, partial η^2^=.079), and the item that assessed whether the condition of brochure and format was boring (*F*_3,122_=2.90, *P*=.04, partial η^2^=.067).

In addition, polynomial contrasts revealed that there was a significant cubic trend for the item of evaluation that assessed whether the condition of brochure and format was informative (*F*_1,122_=6.72, *P*=.01, partial η^2^=.055) and a significant linear trend for the item of evaluation that assessed whether the condition of brochure and format was too long (*F*_1,122_=5.00, *P*=.03, partial η^2^=.048; see Table 4).

When testing for differences among the four conditions, a traditional brochure with a tablet (condition 2) was perceived significantly less positively than a photo story on a tablet (condition 4), as it was harder to understand. Moreover, the traditional brochure shown on paper (condition 1) was also perceived as being less monotonous, less boring, and less lengthy to read in comparison to the traditional brochure with a tablet (condition 2). The traditional brochure with a tablet (condition 2) was also perceived as being significantly more monotonous and less informative than the photo story shown on paper (condition 3).

### Self-Efficacy, Behavioral Intentions, and the Underlying Mechanisms

There was no statistically significant difference in self-efficacy, behavioral intentions, self-referencing, identification and transportation based on the four different conditions (*F*_15,326_=1.18, *P*=.28, Wilk λ=0.86, partial η^2^=.048). However, the findings showed a significant difference among groups (*F*_3,122_=3.44, *P*=.02, partial η^2^=.078) in that the participants deliberately considered that the contents could be related to their own experiences.

**Table 4:** Means and standard deviations of the evaluations of the traditional brochure and photo story in paper and tablet formats in the study (N=126).

“Did you find the booklet...”	Traditional, mean (SD)		Photo story, mean (SD)		*P* value^a^	Multiple comparisons^b^ (conditions)
	Paper (n=32)	Tablet (n=30)	Paper (n=34)	Tablet (n=30)		
Hard to understand?	1.91 (1.17)	2.17 (1.34)	1.65 (1.07)	1.37 (0.77)	.02^c^	2>4
Interesting?	3.69 (1.36)	3.43 (1.07)	3.68 (1.32)	3.57 (1.14)	.54	—^d^
Monotonous?	2.28 (1.25)	3.07 (1.08)	2.26 (1.14)	2.73 (1.14)	.02	2>1&3
Boring?	1.94 (1.11)	2.77 (1.19)	2.15 (1.26)	2.43 (1.14)	.03	2>1
Enjoyable?	2.47 (1.37)	2.37 (1.07)	2.65 (1.28)	2.47 (1.01)	.77	—
Informative?	3.59 (1.54)	3.13 (1.20)	3.94 (1.13)	3.77 (1.14)	.01^c^	3>2
Too long?	1.78 (1.26)	2.50 (1.33)	2.09 (1.22)	2.40 (1.28)	.03	2>1

^a^Statistically significant linear trends based on polynomial contrast.

^b^Only significant multiple comparisons are displayed (*P<*.05).

^c^Statistically significant cubic trend. Otherwise, statistically significant linear trend.

^d^Not significant.

**Table 5:** Means and standard deviations of self-efficacy, behavioral intention, self-referencing, identification, and transportation across four conditions.

Item	Traditional, mean (SD)		Photo story, mean (SD)		Total, mean (SD)
	Paper (n=32)	Tablet (n=30)	Paper (n=34)	Tablet (n=30)	
Self-efficacy	3.98 (0.68)	3.90 (0.75)	3.97 (0.76)	3.88 (0.85)	3.94 (0.75)
Behavioral intention	4.34 (0.53)	4.14 (0.65)	4.32 (0.62)	4.21 (0.75)	4.26 (0.64)
Self-referencing^a^	3.63 (1.01)	2.90 (1.12)	3.05 (1.14)	2.84 (1.05)	3.11 (1.11)
Identification	3.56 (0.72)	3.31 (0.53)	3.61 (0.93)	3.49 (0.85)	3.50 (0.78)
Transportation	2.84 (0.78)	2.67 (0.72)	2.68 (0.76)	2.70 (0.58)	2.72 (0.71)

^a^Significant difference with *P*=.02.

Self-referencing consisted of three items measuring whether the display format of the brochure encouraged participants to think or recall their own experience and reflect on their own conversation with their doctors [22]. In multiple comparisons, the traditional brochure in paper format was significantly the best condition for self-referencing compared to both the traditional brochure and photo story in tablet intervention (see Table 5). It was particularly significant for the item “Did the brochure make you think about yourself and about the conversations with your doctor?” (*F*_3,122_=6.096, *P*=.001, partial η^2^=.130).

For self-efficacy, intention, identification, and transportation, health literacy aspects of the interventions were examined and the results revealed no significant effects of the conditions (*F*_3,122_=0.14, *P*=.94, partial η^2^=.003; *F*_3,122_=0.68, *P*=.57, partial η^2^=.016; *F*_3,122_=0.90, *P*=.44, partial η^2^=.022, and *F*_3,122_=0.43, *P*=.73, partial η^2^=.010, respectively).

## Discussion

### Summary of Main Findings

This study aimed to compare the effectiveness of a photo story intervention presented in different conditions in comparison to a traditional brochure. An evaluation of different forms of patient interventions with 126 older adults in Germany revealed that the photo story intervention was more positively evaluated in comparison to traditional brochures: participants claimed that a photo story is easier to understand and more informative than a traditional brochure. Furthermore, this study found that older adults preferred the paper format to the tablet format due to paper being less monotonous, less boring, and the length of the contents being judged as appropriate.

Regarding older adults’ communicational self-efficacy and behavioral intentions, self-referencing, identification, and transportation, only self-referencing showed significant differences among the different conditions. The participants in the traditional brochure in paper format group (condition 1) scored higher on self-referencing than those in the tablet format for both types of brochure (conditions 2 and 4).

The results that emerged in this study are consistent with the literature that a photo story intervention is an effective communication tool for health information [3,10]. The photo story showed its potential in health behavior application as an encouraging method for individuals to learn certain behaviors by observing a model (in line with Bandura’s social cognitive theory [4]). This effect appeared stronger in the photo story version than in the traditional version. As suggested in previous studies [3,10], the narrative format is not only an uncomplicated and less effortful way to deliver messages through words, but also potentially offers opportunities for self-awareness, reassurance, empathy, and a safe and neutral way to explore the impact of illness in family relationships. This could also be relevant to doctor-patient communication, particularly in primary care consultations when discussing illness, prevention, awareness, and self-care.

Regarding the use of tablets among older adults regardless of the types of brochure, this study showed that participants preferred a paper format, as they found reading a brochure on tablets to be more monotonous, boring, and too lengthy. The results of this study did not corroborate the findings of previous studies [11,24], in which older adults reported more positive than negative attitudes about the technologies they used to obtain health information and the research consent process. Our findings highlight the concern of the potential barriers and challenges older adults face regarding rapid changes in information technology. This is important to keep in mind when translating previous interventions into versions that are meant to be delivered via information technology. Although technology use might generally be considered more effective, this may not be the case for older adults. There is a wide range of explanations for the challenges of using information technology among older adults. For example, lack of instructions and support, lack of clarity in giving instructions and support, lack of knowledge and self-confidence in their personal capabilities to use a tablet, health-related barriers, and high cost of technological equipment [25]. The usage of information technology is also highly influenced by gender, age, (health) literacy, health condition, and educational background [24]. However, solutions are possible. For example, a previous study found that co-creation of interventions using information technology with older adults was appealing and understandable [9]. Such a co-creation approach with interventions like those in this study may help to alleviate some of the challenges faced by the target group.

Considering all conditions in the study, the traditional brochure in tablet format appeared to be the least effective. This can be explained by the preference for the photo story over the traditional brochure, together with the challenges of technology use. Unless the intervention of a photo story was used in tablet format, participants rated reading the traditional brochure on a tablet as hard to understand. This is in line with a previous study, in which older adults in a focus group admitted tablet technology was too complicated and expressed a preference for simpler devices [25]. On the other hand, the participants from the same study expressed the likelihood of using a tablet in the future, and they enjoyed the tablet experience [25]. Therefore, the use of a photo story in health interventions, and the use of technology, are worth investigating further in the future.

Although many positive aspects of technology use have been identified in the literature, the negative aspects of technology use among older adults are also highly relevant. It is especially important to increase the understanding of older adults’ points of view and attitudes toward the use of technology, which is essential to help with introducing technology to this target group and maximizing the potential of technology, particularly to facilitate effective communication in primary care consultations [25]. A previous study showed that older adults recognized that information technology can be discouraging in some ways; therefore, it is essential to have appropriate skills or measures to tackle the difficulties associated with technology use [26].

Although this study did not find significant effects on this photo story intervention regarding participants’ health literacy, communicational self-efficacy, or behavioral intention, it found significant differences among the different conditions regarding self-referencing. This particularly refers to the traditional brochure in paper format (condition 1). This outcome is consistent with findings of a previous study by de Graaf [27]. Older adults might have felt more comfortable with the classic paper format than the electronic device, making it easier to encourage them to think about their personal experiences and conversations with their doctors [27]. There were nonsignificant outcomes for both identification and transportation. For identification, it was more challenging for the participants in this study because of their mean frequencies of doctor visits: most were between every 3 and 6 months, which is perhaps not frequent enough to provoke recognition of the characters as similar or build a social relationship [18]. Most of the participants reported few difficulties in communication with their doctors and few difficulties with transportation, which refers to when participants focused on the events happening in the stories. With fewer relevancies to the story, the participants were less likely to be transported [23].

### Strengths and Limitations

To our knowledge, this study is one of the few studies that investigated the effectiveness of a photo story intervention and the use of tablet technology. The materials were designed and constructed based on the outcomes of pilot studies and focus group discussions, as well as previous studies [19-21]. Conducting a pilot study before the main data collection and analyses ensured that the content was both comprehensible and relevant to the participants, and the reliability and validity of the results was strengthened. One further strength was the randomized assignment of participants to minimize the effect of confounding variables on the systematic variation, thus reducing errors due to measurement or other preventable influences on variation in addition to that of the independent variable [28]. The comparisons of traditional brochures displayed in paper and tablet format were useful in providing additional insights into the health literacy levels of older adults and intervention design for effective communication, especially in primary care consultation. Moreover, the reported outcomes are outlined with the intention to provide an overview of the general trends of the variables among this specific group of participants: older adults aged 50 years and older in clinical settings. This is especially meaningful because this set of variables, to our knowledge, has not yet been widely examined in the literature.

Despite the study having a reasonable sample size, more participants for the different conditions would have been preferable to obtain better power to detect statistical significance [29]. In future studies, in addition to larger sample sizes, longer follow-up measurement points should also be aimed for to enable better intervention checks and analyses of changes over time, as well as collection of more reliable data (eg, objective data from tracking devices). In addition, information technology itself can bring several disadvantages, such as the technical requirements of the users in terms of the need to be technologically health literate, able to read and write, and open to using information technologies and innovations. In addition, user fear of fraud and misuse of their data should be considered. Moreover, devices need to be available and well-functioning, which can be an obstacle for individuals without proper support systems. Another limitation is that the operational definitions of the main variables in the questionnaires are unclear, such as the items of self-efficacy and behavioral intention. Therefore, it is strongly recommended to improve the comprehensiveness of the questionnaire in future studies. Finally, these findings might be specific for the current generation of older adults in Germany and should be investigated with other target groups in different locations.

### Conclusion

In conclusion, when different types of brochure (traditional brochure vs photo story) and display formats (paper vs tablet format) were examined, the photo story and paper formats were found to be effective among older adults aged 50 years and older. Overall, a traditional brochure on a tablet appeared to be the least effective, with higher preference for the photo story instead.

The findings of this study indicate that the use of electronic devices is less helpful for older adults. Several aspects should be taken into consideration in future studies; for example, health literacy, educational background, gender, previous experience with technology, behavioral outcomes, and generational differences to gain further insight into potential influencers [11]. The rise of information technology brings advantages since more people can be addressed and attracted in various fields, such as health care. Different target groups can also be addressed more effectively by tailoring the intervention to their needs.

Information technology can also, however, bring several disadvantages, such as the need to be technologically health literate, able to read and write, and open to using modern technologies. This study highlighted the relevance of some of these aspects with older adults in Germany. These factors should be considered when integrating information/electronic technology into wider situations and populations, globally and individually.

## Appendix

Multimedia Appendix 1 CONSORT‐EHEALTH checklist (V 1.6.1).

## Abbreviations

AURA: Ask, Understand, Remember Assessment
