# Supplementary material for: Testing a Photo Story Intervention in Paper Versus Electronic Tablet Format Compared to a Traditional Brochure Among Older Adults in Germany: Randomized Controlled Trial
Source: JMIR Aging. 2018 Dec 6;1(2):e12145. doi: 10.2196/12145 (PMC6715415; doi:10.2196/12145)
Supplement: Multimedia Appendix 1 [file aging_v1i2e12145_app1.pdf]

CONSORT-EHEALTH (V 1.6.1) - Submission/Publication Form
The CONSORT-EHEALTH checklist is intended for authors of randomized trials evaluating web-based and Internet-based applications/interventions, including mobile interventions, electronic games (incl multiplayer games), social media, certain telehealth applications, and other interactive and/or networked electronic applications. Some of the items (e.g. all subitems under item 5 - description of the intervention) may also be applicable for other study designs.

The goal of the CONSORT EHEALTH checklist and guideline is to be
a) a guide for reporting for authors of RCTs,
b) to form a basis for appraisal of an ehealth trial (in terms of validity)

CONSORT-EHEALTH items/subitems are MANDATORY reporting items for studies published in the Journal of Medical Internet Research and other journals / scientific societies endorsing the checklist.

Items numbered 1., 2., 3., 4a., 4b etc are original CONSORT or CONSORT-NPT (non-pharmacologic treatment) items.
Items with Roman numerals (i., ii, iii, iv etc.) are CONSORT-EHEALTH extensions/clarifications.

As the CONSORT-EHEALTH checklist is still considered in a formative stage, we would ask that you also RATE ON A SCALE OF 1-5 how important/useful you feel each item is FOR THE PURPOSE OF THE CHECKLIST and reporting guideline (optional).

Mandatory reporting items are marked with a red *.
In the textboxes, either copy & paste the relevant sections from your manuscript into this form - please include any quotes from your manuscript in QUOTATION MARKS,
or answer directly by providing additional information not in the manuscript, or elaborating on why the item was not relevant for this study.

YOUR ANSWERS WILL BE PUBLISHED AS A SUPPLEMENTARY

FILE TO YOUR PUBLICATION IN JMIR AND ARE CONSIDERED PART OF YOUR PUBLICATION (IF ACCEPTED).
Please fill in these questions diligently. Information will not be copyedited, so please use proper spelling and grammar, use correct capitalization, and avoid abbreviations.

DO NOT FORGET TO SAVE AS PDF _AND_ CLICK THE SUBMIT BUTTON SO YOUR ANSWERS ARE IN OUR DATABASE !!!

Citation Suggestion (if you append the pdf as Appendix we suggest to cite this paper in the caption):
Eysenbach G, CONSORT-EHEALTH Group
CONSORT-EHEALTH: Improving and Standardizing Evaluation Reports of Web-based and Mobile Health Interventions
J Med Internet Res 2011;13(4):e126
URL: http://www.jmir.org/2011/4/e126/
doi: 10.2196/jmir.1923
PMID: 22209829

* Required
Your name *
First Last

Amanda Whittal

Your answer
Primary Affiliation (short), City, Country *
University of Toronto, Toronto, Canada

Jacobs University Bremen

Your answer
Your e-mail address *
abc@gmail.com

a.whittal@jacobs-universit

Your answer
Title of your manuscript *
Provide the (draft) title of your manuscript.
Your answer

Testing
a photo novel intervention in paper versus electronic tablet format,
and in comparison to a traditional brochure among

Name of your App/Software/Intervention *
If there is a short and a long/alternate name, write the short name first and add the long name in brackets.

Photo novel

Your answer
Evaluated Version (if any)
e.g. "V1", "Release 2017-03-01", "Version 2.0.27913"

N/A

Your answer
Language(s) *
What language is the intervention/app in? If multiple languages are available, separate by comma (e.g. "English, French")

German

Your answer
URL of your Intervention Website or App
e.g. a direct link to the mobile app on app in appstore (itunes, Google Play), or URL of the website. If the intervention is a DVD or hardware, you can also link to an Amazon page.

Your answer
URL of an image/screenshot (optional)

Your answer
Accessibility *
Can an enduser access the intervention presently?
access is free and open
access only for special usergroups, not open
access is open to everyone, but requires payment/subscription/in-app purchases
app/intervention no longer accessible
Other:

Primary Medical Indication/Disease/Condition *
e.g. "Stress", "Diabetes", or define the target group in brackets after the condition, e.g. "Autism (Parents of children with)", "Alzheimers (Informal Caregivers of)"

Not applicable

Your answer

Primary Outcomes measured in trial *
comma-separated list of primary outcomes reported in the trial

Health literacy and commu

Your answer

Secondary/other outcomes
Are there any other outcomes the intervention is expected to affect?
Your answer

Recommended "Dose" *
What do the instructions for users say on how often the app should be used?

Approximately Daily
Approximately Weekly
Approximately Monthly
Approximately Yearly
"as needed"
Other:

Approx. Percentage of Users (starters) still using the app as recommended after 3 months *

unknown / not evaluated
0-10%
11-20%
21-30%
31-40%
41-50%
51-60%
61-70%

71%-80%
81-90%
91-100%
Other:

Overall, was the app/intervention effective? *
yes: all primary outcomes were significantly better in intervention group vs control
partly: SOME primary outcomes were significantly better in intervention group vs control
no statistically significant difference between control and intervention
potentially harmful: control was significantly better than intervention in one or more outcomes
inconclusive: more research is needed
Other:

Article Preparation Status/Stage *
At which stage in your article preparation are you currently (at the time you fill in this form)
not submitted yet - in early draft status
not submitted yet - in late draft status, just before submission
submitted to a journal but not reviewed yet
submitted to a journal and after receiving initial reviewer comments
submitted to a journal and accepted, but not published yet
published
Other:

Journal *
If you already know where you will submit this paper (or if it is already submitted), please provide the journal name (if it is not JMIR, provide the journal name under "other")
not submitted yet / unclear where I will submit this
Journal of Medical Internet Research (JMIR)
JMIR mHealth and UHealth
JMIR Serious Games
JMIR Mental Health

JMIR Public Health
JMIR Formative Research
Other JMIR sister journal
Other:

Is this a full powered effectiveness trial or a pilot/feasibility trial? *
Pilot/feasibility
Fully powered
Manuscript tracking number *
If this is a JMIR submission, please provide the manuscript tracking number under "other" (The ms tracking number can be found in the submission acknowledgement email, or when you login as author in JMIR. If the paper is already published in JMIR, then the ms tracking number is the four-digit number at the end of the DOI, to be found at the bottom of each published article in JMIR)
no ms number (yet) / not (yet) submitted to / published in JMIR
Other:
12145

TITLE AND ABSTRACT

1a) TITLE: Identification as a randomized trial in the title

1a) Does your paper address CONSORT item 1a? *
I.e does the title contain the phrase "Randomized Controlled Trial"? (if not, explain the reason under "other")
yes
Other:

1a-i) Identify the mode of delivery in the title
Identify the mode of delivery. Preferably use “web-based” and/or “mobile” and/or “electronic game” in the title. Avoid ambiguous terms like “online”, “virtual”, “interactive”. Use “Internet-based” only if

Intervention includes non-web-based Internet components (e.g. email), use “computer-based” or “electronic” only if offline products are used. Use “virtual” only in the context of “virtual reality” (3-D worlds). Use “online” only in the context of “online support groups”. Complement or substitute product names with broader terms for the class of products (such as “mobile” or “smart phone” instead of “iphone”), especially if the application runs on different platforms.
subitem not at all important
1
2
3
4
5
essential
Does your paper address subitem 1a-i? *
Copy and paste relevant sections from manuscript title (include quotes in quotation marks "like this" to indicate direct quotes from your manuscript), or elaborate on this item by providing additional information not in the ms, or briefly explain why the item is not applicable/relevant for your study
Your answer
"Testing a photo novel intervention in paper versus
1a-ii) Non-web-based components or important co-interventions in title
Mention non-web-based components or important co-interventions in title, if any (e.g., “with telephone support”).
subitem not at all important
1
2
3
4
5
essential
Does your paper address subitem 1a-ii?
Copy and paste relevant sections from manuscript title (include quotes in quotation marks "like this" to indicate direct quotes from your manuscript), or elaborate on this item by providing additional

information not in the ms, or briefly explain why the item is not applicable/relevant for your study

Your answer

"...in comparison to a traditional brochure among

1a-iii) Primary condition or target group in the title

Mention primary condition or target group in the title, if any (e.g., “for children with Type I Diabetes”) Example: A Web-based and Mobile Intervention with Telephone Support for Children with Type I Diabetes: Randomized Controlled Trial

subitem not at all important

1

2

3

4

5

essential

Does your paper address subitem 1a-iii? *

Copy and paste relevant sections from manuscript title (include quotes in quotation marks "like this" to indicate direct quotes from your manuscript), or elaborate on this item by providing additional information not in the ms, or briefly explain why the item is not applicable/relevant for your study

Your answer

"...among older adults in Germany..."

1b) ABSTRACT: Structured summary of trial design, methods, results, and conclusions

NPT extension: Description of experimental treatment, comparator, care providers, centers, and blinding status.
1b-i) Key features/functionalities/components of the intervention and comparator in the METHODS section of the ABSTRACT
Mention key features/functionalities/components of the intervention and comparator in the abstract. If possible, also mention theories and principles used for designing the site. Keep in mind the needs of systematic reviewers and indexers by including important synonyms. (Note: Only report in the abstract what the main paper is reporting. If this information is missing from the main body of text, consider adding it)

subitem not at all important
1
2
3
4
5
essential
Does your paper address subitem 1b-i? *
Copy and paste relevant sections from the manuscript abstract (include quotes in quotation marks "like this" to indicate direct quotes from your manuscript), or elaborate on this item by providing additional information not in the ms, or briefly explain why the item is not applicable/relevant for your study
Your answer

"...randomly
assigned to one of four
intervention conditions:
Condition
1—traditional brochure in
paper format, Condition 2—
photo novel in paper
format, Condition 3—
traditional brochure in tablet
format, and
Condition 4—photo novel in

1b-ii) Level of human involvement in the METHODS section of the ABSTRACT
Clarify the level of human involvement in the abstract, e.g., use phrases like “fully automated” vs. “therapist/nurse/care provider/physician-assisted” (mention number and expertise of providers involved, if any). (Note: Only report in the abstract what the main paper is reporting. If this information is missing from the main body of text, consider adding it)
subitem not at all important
1
2
3
4
5

essential
Does your paper address subitem 1b-ii?
Copy and paste relevant sections from the manuscript abstract (include quotes in quotation marks "like this" to indicate direct quotes from your manuscript), or elaborate on this item by providing additional information not in the ms, or briefly explain why the item is not applicable/relevant for your study
Your answer

"...approached
and recruited by researchers
and responsible people from
senior dav

1b-iii) Open vs. closed, web-based (self-assessment) vs. face-to-face assessments in the METHODS section of the ABSTRACT
Mention how participants were recruited (online vs. offline), e.g., from an open access website or from a clinic or a closed online user group (closed usergroup trial), and clarify if this was a purely web-based trial, or there were face-to-face components (as part of the intervention or for assessment). Clearly say if outcomes were self-assessed through questionnaires (as common in web-based trials). Note: In traditional offline trials, an open trial (open-label trial) is a type of clinical trial in which both the researchers and participants know which treatment is being administered. To avoid confusion, use “blinded” or “unblinded” to indicated the level of blinding instead of “open”, as “open” in web-based trials usually refers to “open access” (i.e. participants can self-enrol). (Note: Only report in the abstract what the main paper is reporting. If this information is missing from the main body of text, consider adding it)
subitem not at all important
1
2
3
4
5
essential
Does your paper address subitem 1b-iii?
Copy and paste relevant sections from the manuscript abstract (include

quotes in quotation marks "like this" to indicate direct quotes from your manuscript), or elaborate on this item by providing additional information not in the ms, or briefly explain why the item is not applicable/relevant for your study

Your answer

"...This study used open and face-to-face assessment

1b-iv) RESULTS section in abstract must contain use data

Report number of participants enrolled/assessed in each group, the use/uptake of the intervention (e.g., attrition/adherence metrics, use over time, number of logins etc.), in addition to primary/secondary outcomes. (Note: Only report in the abstract what the main paper is reporting. If this information is missing from the main body of text, consider adding it)

subitem not at all important

1

2

3

4

5

essential

Does your paper address subitem 1b-iv?

Copy and paste relevant sections from the manuscript abstract (include quotes in quotation marks "like this" to indicate direct quotes from your manuscript), or elaborate on this item by providing additional information not in the ms, or briefly explain why the item is not applicable/relevant for your study

Your answer

"In
 comparison to the traditional brochure, the photo novel was considered
by the study participants to be easier to understand, t(124) = 2.62, P =
 .01,more enjoyable, t(124) = -0.68, P = .05, and more informative,
t(124) = -2.17, P = .05 by the study participants. Specifically,
participants preferred the paper format because they found it less
monotonous, t(124) = -3.05, P = .003; less boring, t(124) = -2.65, P =
.009; and not too long, t(124) = -2.26, P = .03, compared to the tablet
format. Among all conditions, the traditional brochure with a tablet
(Condition 2) was also perceived as more monotonous (M = 3.07; SD = 1.08), boring (M = 2.77; SD = 1.19), and too long to read(M = 2.50; SD =
[illegible]

1b-v) CONCLUSIONS/DISCUSSION in abstract for negative trials

Conclusions/Discussions in abstract for negative trials: Discuss the primary outcome - if the trial is negative (primary outcome not changed), and the intervention was not used, discuss whether negative results are attributable to lack of uptake and discuss reasons. (Note: Only report in the abstract what the main paper is reporting. If this information is missing from the main body of text, consider adding it)

subitem not at all important

1

2

3

4

5

essential

Does your paper address subitem 1b-v?

Copy and paste relevant sections from the manuscript abstract (include quotes in quotation marks "like this" to indicate direct quotes from your manuscript), or elaborate on this item by providing additional information not in the ms, or briefly explain why the item is not applicable/relevant for your study

Your answer

"Traditional
brochures on a tablet seem to be the least effective in primary care
consultations among all conditions of types of brochure and display
format, which is the main

INTRODUCTION

2a) In INTRODUCTION: Scientific background and explanation of rationale

2a-i) Problem and the type of system/solution

Describe the problem and the type of system/solution that is object of the study: intended as stand-alone intervention vs. incorporated in broader health care program? Intended for a particular patient population? Goals of the intervention, e.g., being more cost-effective to other interventions, replace or complement other solutions? (Note: Details about the intervention are provided in “Methods” under 5)

subitem not at all important

1

2

3

4
5
essential
Does your paper address subitem 2a-i? *
Copy and paste relevant sections from the manuscript (include quotes in quotation marks "like this" to indicate direct quotes from your manuscript), or elaborate on this item by providing additional information not in the ms, or briefly explain why the item is not applicable/relevant for your study
Your answer

"Despite
the wide range of studies available, there is a visible lack of
research, especially randomized controlled trials (RCTs) with study
[illegible]

2a-ii) Scientific background, rationale: What is known about the (type of) system
Scientific background, rationale: What is known about the (type of) system that is the object of the study (be sure to discuss the use of similar systems for other conditions/diagnoses, if appropiate), motivation for the study, i.e. what are the reasons for and what is the context for this specific study, from which stakeholder viewpoint is the study performed, potential impact of findings [2]. Briefly justify the choice of the comparator.
subitem not at all important
1
2
3
4
5
essential
Does your paper address subitem 2a-ii? *
Copy and paste relevant sections from the manuscript (include quotes in quotation marks "like this" to indicate direct quotes from your manuscript), or elaborate on this item by providing additional information not in the ms, or briefly explain why the item is not

applicable/relevant for your study
Your answer

"Narrative
 communication as a tool has been strongly recommended in previous
studies [3], as it is considered to be a basic approach of human interaction, regardless of literacy level. Photo Novel(or picture story)
 is an alternative tool in a [illegible]

2b) In INTRODUCTION: Specific objectives or hypotheses

Does your paper address CONSORT subitem 2b? *

Copy and paste relevant sections from the manuscript (include quotes in quotation marks "like this" to indicate direct quotes from your manuscript), or elaborate on this item by providing additional information not in the ms, or briefly explain why the item is not applicable/relevant for your study

Your answer

"i.
Which intervention do older adults appreciate more: the photo novel or
the traditional brochure?
ii. Which format do older adults prefer in the intervention: the paper format or the tablet format?
iii. Which condition of the interventions (the photo novel or the
traditional brochure provided on paper or on a tablet) is more effective
in increasing older adults' communicational self-

METHODS

3a) Description of trial design (such as parallel, factorial) including allocation ratio

Does your paper address CONSORT subitem 3a? *
Copy and paste relevant sections from the manuscript (include quotes in quotation marks "like this" to indicate direct quotes from your manuscript), or elaborate on this item by providing additional information not in the ms, or briefly explain why the item is not applicable/relevant for your study
Your answer

"A
total of 126 participants,
ages 50 and older (M=71.85,
SD=10.13), were
recruited and randomly
assigned to one of four test

3b) Important changes to methods after trial commencement (such as eligibility criteria), with reasons

Does your paper address CONSORT subitem 3b? *

Copy and paste relevant sections from the manuscript (include quotes in quotation marks "like this" to indicate direct quotes from your manuscript), or elaborate on this item by providing additional

information not in the ms, or briefly explain why the item is not applicable/relevant for your study

Your answer

This item is not applicable as there were no changes after

3b-i) Bug fixes, Downtimes, Content Changes

Bug fixes, Downtimes, Content Changes: ehealth systems are often dynamic systems. A description of changes to methods therefore also includes important changes made on the intervention or comparator during the trial (e.g., major bug fixes or changes in the functionality or content) (5-iii) and other “unexpected events” that may have influenced study design such as staff changes, system failures/downtimes, etc. [2].

subitem not at all important

1

2

3

4

5

essential

Does your paper address subitem 3b-i?

Copy and paste relevant sections from the manuscript (include quotes in quotation marks "like this" to indicate direct quotes from your manuscript), or elaborate on this item by providing additional information not in the ms, or briefly explain why the item is not applicable/relevant for your study

Your answer

As this was a one time trial, there was no need for bug fixes, and ere downtimes or content changes

4a) Eligibility criteria for participants

Does your paper address CONSORT subitem 4a? *
Copy and paste relevant sections from the manuscript (include quotes in quotation marks "like this" to indicate direct quotes from your manuscript), or elaborate on this item by providing additional information not in the ms, or briefly explain why the item is not applicable/relevant for your study
Your answer

Participants
had to be older adults.
"Participants were
approached and recruited by
researchers via responsible
people of senior day care and
rehabilitation
centres in Germany, as well

4a-i) Computer / Internet literacy

Computer / Internet literacy is often an implicit “de facto” eligibility criterion - this should be explicitly clarified.

subitem not at all important

1

2

3

4

5

essential

Does your paper address subitem 4a-i?

Copy and paste relevant sections from the manuscript (include quotes in quotation marks "like this" to indicate direct quotes from your manuscript), or elaborate on this item by providing additional information not in the ms, or briefly explain why the item is not applicable/relevant for your study

Your answer

There was no basic level of
computer literacy assessed

4a-ii) Open vs. closed, web-based vs. face-to-face assessments:

Open vs. closed, web-based vs. face-to-face assessments: Mention how participants were recruited (online vs. offline), e.g., from an open access website or from a clinic, and clarify if this was a purely web-based trial, or there were face-to-face components (as part of the intervention or for assessment), i.e., to what degree got the study team to know the participant. In online-only trials, clarify if participants were quasi-anonymous and whether having multiple identities was possible or whether technical or logistical measures (e.g., cookies, email confirmation, phone calls) were used to detect/prevent these.

subitem not at all important

1

2
3
4
5
essential
Does your paper address subitem 4a-ii? *
Copy and paste relevant sections from the manuscript (include quotes in quotation marks "like this" to indicate direct quotes from your manuscript), or elaborate on this item by providing additional information not in the ms, or briefly explain why the item is not applicable/relevant for your study
Your answer

"This study used open and
face-to-face assessment

4a-iii) Information giving during recruitment
Information given during recruitment. Specify how participants were briefed for recruitment and in the informed consent procedures (e.g., publish the informed consent documentation as appendix, see also item X26), as this information may have an effect on user self-selection, user expectation and may also bias results.
subitem not at all important
1
2
3
4
5
essential
Does your paper address subitem 4a-iii?
Copy and paste relevant sections from the manuscript (include quotes in quotation marks "like this" to indicate direct quotes from your manuscript), or elaborate on this item by providing additional information not in the ms, or briefly explain why the item is not applicable/relevant for your study
Your answer

"The
 researchers introduced
themselves to participants
and provided
information sheets, which
contained both the aim and
basic information
about the current study...
After signing the consent
form, each
participant received a
[illegible]

4b) Settings and locations where the data were collected

Does your paper address CONSORT subitem 4b? *

Copy and paste relevant sections from the manuscript (include quotes in quotation marks "like this" to indicate direct quotes from your manuscript), or elaborate on this item by providing additional information not in the ms, or briefly explain why the item is not applicable/relevant for your study

Your answer

"Participants
were approached and recruited by researchers via responsible people of senior day care and rehabilitation centres in

4b-i) Report if outcomes were (self-)assessed through online questionnaires

Clearly report if outcomes were (self-)assessed through online questionnaires (as common in web-based trials) or otherwise.

subitem not at all important

1

2

3

4

5

essential

Does your paper address subitem 4b-i? *

Copy and paste relevant sections from the manuscript (include quotes in quotation marks "like this" to indicate direct quotes from your manuscript), or elaborate on this item by providing additional information not in the ms, or briefly explain why the item is not applicable/relevant for your study

Your answer

"To
evaluate the effectiveness, participants were asked to fill out
questionnaires before and after the intervention to

4b-ii) Report how institutional affiliations are displayed

Report how institutional affiliations are displayed to potential participants [on ehealth media], as affiliations with prestigious hospitals or universities may affect volunteer rates, use, and reactions with

regards to an intervention.(Not a required item – describe only if this may bias results)
subitem not at all important
1
2
3
4
5
essential
Does your paper address subitem 4b-ii?
Copy and paste relevant sections from the manuscript (include quotes in quotation marks "like this" to indicate direct quotes from your manuscript), or elaborate on this item by providing additional information not in the ms, or briefly explain why the item is not applicable/relevant for your study
Your answer

"Participants
were approached and recruited by researchers via responsible people of senior day care and rehabilitation centres in Germany, as well as sports clubs or meeting places of Aktive Menschen Bremen e.V. [illegible]

5) The interventions for each group with sufficient details to allow replication, including how and when they were actually administered

5-i) Mention names, credential, affiliations of the developers, sponsors, and owners

Mention names, credential, affiliations of the developers, sponsors, and owners [6] (if authors/evaluators are owners or developer of the software, this needs to be declared in a “Conflict of interest” section or mentioned elsewhere in the manuscript).

subitem not at all important

1

2

3

4

5
essential
Does your paper address subitem 5-i?
Copy and paste relevant sections from the manuscript (include quotes in quotation marks "like this" to indicate direct quotes from your manuscript), or elaborate on this item by providing additional information not in the ms, or briefly explain why the item is not applicable/relevant for your study
Your answer

"The photo novels and brochures used in this study were developed by Elco van der Meer..."

5-ii) Describe the history/development process
Describe the history/development process of the application and previous formative evaluations (e.g., focus groups, usability testing), as these will have an impact on adoption/use rates and help with interpreting results.
subitem not at all important
1
2
3
4
5
essential
Does your paper address subitem 5-ii?
Copy and paste relevant sections from the manuscript (include quotes in quotation marks "like this" to indicate direct quotes from your manuscript), or elaborate on this item by providing additional information not in the ms, or briefly explain why the item is not applicable/relevant for your study
Your answer

"...based
on both previous literature [3,4,6] and the outcomes of focus group

5-iii) Revisions and updating
Revisions and updating. Clearly mention the date and/or version

number of the application/intervention (and comparator, if applicable) evaluated, or describe whether the intervention underwent major changes during the evaluation process, or whether the development and/or content was "frozen" during the trial. Describe dynamic components such as news feeds or changing content which may have an impact on the replicability of the intervention (for unexpected events see item 3b).

subitem not at all important

1

2

3

4

5

essential

Does your paper address subitem 5-iii?

Copy and paste relevant sections from the manuscript (include quotes in quotation marks "like this" to indicate direct quotes from your manuscript), or elaborate on this item by providing additional information not in the ms, or briefly explain why the item is not applicable/relevant for your study

Your answer

There was not an updated version used in this study

5-iv) Quality assurance methods

Provide information on quality assurance methods to ensure accuracy and quality of information provided [1], if applicable.

subitem not at all important

1

2

3

4

5

essential

Does your paper address subitem 5-iv?

Copy and paste relevant sections from the manuscript (include quotes in quotation marks "like this" to indicate direct quotes from your manuscript), or elaborate on this item by providing additional

information not in the ms, or briefly explain why the item is not applicable/relevant for your study
Your answer

"To
 guarantee a standardised approach, all participating researchers

5-v) Ensure replicability by publishing the source code, and/or providing screenshots/screen-capture video, and/or providing flowcharts of the algorithms used
Ensure replicability by publishing the source code, and/or providing screenshots/screen-capture video, and/or providing flowcharts of the algorithms used. Replicability (i.e., other researchers should in principle be able to replicate the study) is a hallmark of scientific reporting.
subitem not at all important
1
2
3
4
5
essential
Does your paper address subitem 5-v?
Copy and paste relevant sections from the manuscript (include quotes in quotation marks "like this" to indicate direct quotes from your manuscript), or elaborate on this item by providing additional information not in the ms, or briefly explain why the item is not applicable/relevant for your study
Your answer

There is not a source code for this intervention

5-vi) Digital preservation
Digital preservation: Provide the URL of the application, but as the intervention is likely to change or disappear over the course of the years; also make sure the intervention is archived (Internet Archive, webcitation.org, and/or publishing the source code or screenshots/videos alongside the article). As pages behind login screens cannot be archived, consider creating demo pages which are accessible without login.

subitem not at all important
1
2
3
4
5
essential
Does your paper address subitem 5-vi?
Copy and paste relevant sections from the manuscript (include quotes in quotation marks "like this" to indicate direct quotes from your manuscript), or elaborate on this item by providing additional information not in the ms, or briefly explain why the item is not applicable/relevant for your study
Your answer

There is not a URL for this intervention

5-vii) Access
Access: Describe how participants accessed the application, in what setting/context, if they had to pay (or were paid) or not, whether they had to be a member of specific group. If known, describe how participants obtained “access to the platform and Internet” [1]. To ensure access for editors/reviewers/readers, consider to provide a “backdoor” login account or demo mode for reviewers/readers to explore the application (also important for archiving purposes, see vi).
subitem not at all important
1
2
3
4
5
essential
Does your paper address subitem 5-vii? *
Copy and paste relevant sections from the manuscript (include quotes in quotation marks "like this" to indicate direct quotes from your manuscript), or elaborate on this item by providing additional information not in the ms, or briefly explain why the item is not applicable/relevant for your study

Your answer

"After
 signing the consent form,
each participant received a
questionnaire and
 either the traditional
brochure or photo novel in a

5-viii) Mode of delivery, features/functionalities/components of the intervention and comparator, and the theoretical framework

Describe mode of delivery, features/functionalities/components of the intervention and comparator, and the theoretical framework [6] used to design them (instructional strategy [1], behaviour change techniques, persuasive features, etc., see e.g., [7, 8] for terminology). This includes an in-depth description of the content (including where it is coming from and who developed it) [1]," whether [and how] it is tailored to individual circumstances and allows users to track their progress and receive feedback" [6]. This also includes a description of communication delivery channels and – if computer-mediated communication is a component – whether communication was synchronous or asynchronous [6]. It also includes information on presentation strategies [1], including page design principles, average amount of text on pages, presence of hyperlinks to other resources, etc. [1].

subitem not at all important

1

2

3

4

5

essential

Does your paper address subitem 5-viii? *

Copy and paste relevant sections from the manuscript (include quotes in quotation marks "like this" to indicate direct quotes from your manuscript), or elaborate on this item by providing additional information not in the ms, or briefly explain why the item is not applicable/relevant for your study

Your answer

"The
 intervention was developed by partners of the IROHLA (Intervention
Research on Health Literacy among the Ageing
Population) consortium to improve older adults' health literacy and communication skills during
care consultations..."
"Participants in the two tablet conditions were

5-ix) Describe use parameters

Describe use parameters (e.g., intended “doses” and optimal timing for use). Clarify what instructions or recommendations were given to the user, e.g., regarding timing, frequency, heaviness of use, if any, or was the intervention used ad libitum.

subitem not at all important

1

2

3

4

5

essential

Does your paper address subitem 5-ix?

Copy and paste relevant sections from the manuscript (include quotes in quotation marks "like this" to indicate direct quotes from your manuscript), or elaborate on this item by providing additional information not in the ms, or briefly explain why the item is not applicable/relevant for your study

Your answer

There
 were not specified parameters, participants were shown the different versions and asked for their
preference and assessed on

5-x) Clarify the level of human involvement

Clarify the level of human involvement (care providers or health professionals, also technical assistance) in the e-intervention or as co-

intervention (detail number and expertise of professionals involved, if any, as well as “type of assistance offered, the timing and frequency of the support, how it is initiated, and the medium by which the assistance is delivered”. It may be necessary to distinguish between the level of human involvement required for the trial, and the level of human involvement required for a routine application outside of a RCT setting (discuss under item 21 – generalizability).
subitem not at all important
1
2
3
4
5
essential
Does your paper address subitem 5-x?
Copy and paste relevant sections from the manuscript (include quotes in quotation marks "like this" to indicate direct quotes from your manuscript), or elaborate on this item by providing additional information not in the ms, or briefly explain why the item is not applicable/relevant for your study
Your answer
"After
signing the consent form,
each participant received a
questionnaire and
either the traditional
brochure or photo novel in a
5-xi) Report any prompts/reminders used
Report any prompts/reminders used: Clarify if there were prompts (letters, emails, phone calls, SMS) to use the application, what triggered them, frequency etc. It may be necessary to distinguish between the level of prompts/reminders required for the trial, and the level of prompts/reminders for a routine application outside of a RCT setting (discuss under item 21 – generalizability).
subitem not at all important
1
2
3

4
5
essential
Does your paper address subitem 5-xi? *
Copy and paste relevant sections from the manuscript (include quotes in quotation marks "like this" to indicate direct quotes from your manuscript), or elaborate on this item by providing additional information not in the ms, or briefly explain why the item is not applicable/relevant for your study
Your answer

There were no prompts or reminders as it was a one

5-xii) Describe any co-interventions (incl. training/support)
Describe any co-interventions (incl. training/support): Clearly state any interventions that are provided in addition to the targeted eHealth intervention, as ehealth intervention may not be designed as stand-alone intervention. This includes training sessions and support [1]. It may be necessary to distinguish between the level of training required for the trial, and the level of training for a routine application outside of a RCT setting (discuss under item 21 – generalizability.
subitem not at all important
1
2
3
4
5
essential
Does your paper address subitem 5-xii? *
Copy and paste relevant sections from the manuscript (include quotes in quotation marks "like this" to indicate direct quotes from your manuscript), or elaborate on this item by providing additional information not in the ms, or briefly explain why the item is not applicable/relevant for your study
Your answer

There were no co-interventions

6a) Completely defined pre-specified primary and secondary outcome

measures, including how and when they were assessed

Does your paper address CONSORT subitem 6a? *
Copy and paste relevant sections from the manuscript (include quotes in quotation marks "like this" to indicate direct quotes from your manuscript), or elaborate on this item by providing additional information not in the ms, or briefly explain why the item is not applicable/relevant for your study
Your answer

"The
questionnaire consisted of two parts: The first part contained
questions regarding socio-demographics, perceived health and well-being measurements, morbidities and the frequency of GP consultations.
Further, the Health Literacy Set of Brief Screening Questions (SBSQ) was
utilised..." "In the second part, questions related to each story of
the traditional brochure, including domain-specific self-efficacies
(e.g., 'You have the feeling

6a-i) Online questionnaires: describe if they were validated for online use and apply CHERRIES items to describe how the questionnaires were designed/deployed

If outcomes were obtained through online questionnaires, describe if they were validated for online use and apply CHERRIES items to describe how the questionnaires were designed/deployed [9].

subitem not at all important

1

2

3

4

5

essential

Does your paper address subitem 6a-i?

Copy and paste relevant sections from manuscript text

Your answer

Online questionnaires were not used

6a-ii) Describe whether and how "use" (including intensity of use/dosage) was defined/measured/monitored

Describe whether and how "use" (including intensity of use/dosage) was defined/measured/monitored (logins, logfile analysis, etc.).

Use/adoption metrics are important process outcomes that should be reported in any ehealth trial.
subitem not at all important
1
2
3
4
5
essential
Does your paper address subitem 6a-ii?
Copy and paste relevant sections from manuscript text
Your answer

It was a one time use only

6a-iii) Describe whether, how, and when qualitative feedback from participants was obtained
Describe whether, how, and when qualitative feedback from participants was obtained (e.g., through emails, feedback forms, interviews, focus groups).
subitem not at all important
1
2
3
4
5
essential
Does your paper address subitem 6a-iii?
Copy and paste relevant sections from manuscript text
Your answer

This was a quantitative study

6b) Any changes to trial outcomes after the trial commenced, with reasons

Does your paper address CONSORT subitem 6b? *
Copy and paste relevant sections from the manuscript (include quotes in quotation marks "like this" to indicate direct quotes from your manuscript), or elaborate on this item by providing additional information not in the ms, or briefly explain why the item is not applicable/relevant for your study

Your answer

No changes were made

7a) How sample size was determined

NPT: When applicable, details of whether and how the clustering by care provides or centers was addressed
7a-i) Describe whether and how expected attrition was taken into account when calculating the sample size
Describe whether and how expected attrition was taken into account when calculating the sample size.
subitem not at all important
1
2
3
4

5
essential
Does your paper address subitem 7a-i?
Copy and paste relevant sections from manuscript title (include quotes in quotation marks "like this" to indicate direct quotes from your manuscript), or elaborate on this item by providing additional information not in the ms, or briefly explain why the item is not applicable/relevant for your study
Your answer

It was a one time trial, there was no attrition problem

7b) When applicable, explanation of any interim analyses and stopping guidelines

Does your paper address CONSORT subitem 7b? *

Copy and paste relevant sections from the manuscript (include quotes in quotation marks "like this" to indicate direct quotes from your manuscript), or elaborate on this item by providing additional information not in the ms, or briefly explain why the item is not applicable/relevant for your study

Your answer

N/A

8a) Method used to generate the random allocation sequence

NPT: When applicable, how care providers were allocated to each trial group

Does your paper address CONSORT subitem 8a? *

Copy and paste relevant sections from the manuscript (include quotes in quotation marks "like this" to indicate direct quotes from your manuscript), or elaborate on this item by providing additional information not in the ms, or briefly explain why the item is not applicable/relevant for your study

Your answer

"...were recruited and randomly assigned to one of four test conditions before they were approached by a

8b) Type of randomisation; details of any restriction (such as blocking and block size)

Does your paper address CONSORT subitem 8b? *

Copy and paste relevant sections from the manuscript (include quotes in quotation marks "like this" to indicate direct quotes from your manuscript), or elaborate on this item by providing additional information not in the ms, or briefly explain why the item is not applicable/relevant for your study

Your answer

N/A, it was a simple evenly distributed random allocation

9) Mechanism used to implement the random allocation sequence (such as sequentially numbered containers), describing any steps taken to conceal the sequence until interventions were assigned

Does your paper address CONSORT subitem 9? *

Copy and paste relevant sections from the manuscript (include quotes in quotation marks "like this" to indicate direct quotes from your manuscript), or elaborate on this item by providing additional information not in the ms, or briefly explain why the item is not

applicable/relevant for your study
Your answer

N/A, it was a simple evenly distributed random allocation

10) Who generated the random allocation sequence, who enrolled participants, and who assigned participants to interventions

Does your paper address CONSORT subitem 10? *
Copy and paste relevant sections from the manuscript (include quotes in quotation marks "like this" to indicate direct quotes from your manuscript), or elaborate on this item by providing additional information not in the ms, or briefly explain why the item is not

applicable/relevant for your study

Your answer

"...were recruited and randomly assigned to one of four test conditions before they were approached by a

11a) If done, who was blinded after assignment to interventions (for example, participants, care providers, those assessing outcomes) and how

NPT: Whether or not administering co-interventions were blinded to group assignment

11a-i) Specify who was blinded, and who wasn’t

Specify who was blinded, and who wasn't. Usually, in web-based trials it is not possible to blind the participants [1, 3] (this should be clearly acknowledged), but it may be possible to blind outcome assessors, those doing data analysis or those administering co-interventions (if any).
subitem not at all important
1
2
3
4
5
essential
Does your paper address subitem 11a-i? *
Copy and paste relevant sections from the manuscript (include quotes in quotation marks "like this" to indicate direct quotes from your manuscript), or elaborate on this item by providing additional information not in the ms, or briefly explain why the item is not applicable/relevant for your study
Your answer
N/A - no one was blinded
11a-ii) Discuss e.g., whether participants knew which intervention was the "intervention of interest" and which one was the "comparator"
Informed consent procedures (4a-ii) can create biases and certain expectations - discuss e.g., whether participants knew which intervention was the "intervention of interest" and which one was the "comparator".
subitem not at all important
1
2
3
4
5
essential
Does your paper address subitem 11a-ii?
Copy and paste relevant sections from the manuscript (include quotes in quotation marks "like this" to indicate direct quotes from your

manuscript), or elaborate on this item by providing additional information not in the ms, or briefly explain why the item is not applicable/relevant for your study

Your answer

All conditions were of interest

11b) If relevant, description of the similarity of interventions

(this item is usually not relevant for ehealth trials as it refers to similarity of a placebo or sham intervention to a active medication/intervention)

Does your paper address CONSORT subitem 11b? *

Copy and paste relevant sections from the manuscript (include quotes in quotation marks "like this" to indicate direct quotes from your manuscript), or elaborate on this item by providing additional information not in the ms, or briefly explain why the item is not applicable/relevant for your study

Your answer

"They
 were randomly assigned to
one of four intervention
conditions:
Condition 1—traditional
brochure in paper format,
Condition 2—photo
[illegible]

12a) Statistical methods used to compare groups for primary and secondary outcomes

NPT: When applicable, details of whether and how the clustering by care providers or centers was addressed

Does your paper address CONSORT subitem 12a? *

Copy and paste relevant sections from the manuscript (include quotes in quotation marks "like this" to indicate direct quotes from your manuscript), or elaborate on this item by providing additional information not in the ms, or briefly explain why the item is not applicable/relevant for your study

Your answer

"...independent
t-test with seven
evaluationvariables was
conducted to compare the
means of the two
independent groups; photo
novel (N=64) and
traditionalbrochure
(N=62).Another independent
t-test was conducted to
examine the mean
differences between paper
(N=66) and tablet formats
(N=60). In addition, an
Analysis of Variance (ANOVA)
was performed to
gain insight into potential
differences among the four
conditions
regarding the traditional
[illegible]

12a-i) Imputation techniques to deal with attrition / missing values
Imputation techniques to deal with attrition / missing values: Not all participants will use the intervention/comparator as intended and attrition is typically high in ehealth trials. Specify how participants who did not use the application or dropped out from the trial were treated in the statistical analysis (a complete case analysis is strongly discouraged, and simple imputation techniques such as LOCF may also be problematic [4]).
subitem not at all important
1
2
3
4
5
essential
Does your paper address subitem 12a-i? *
Copy and paste relevant sections from the manuscript (include quotes in quotation marks "like this" to indicate direct quotes from your manuscript), or elaborate on this item by providing additional information not in the ms, or briefly explain why the item is not applicable/relevant for your study

Your answer

No imputation was done

12b) Methods for additional analyses, such as subgroup analyses and adjusted analyses

Does your paper address CONSORT subitem 12b? *

Copy and paste relevant sections from the manuscript (include quotes in quotation marks "like this" to indicate direct quotes from your manuscript), or elaborate on this item by providing additional information not in the ms, or briefly explain why the item is not applicable/relevant for your study

Your answer

No adjusted analyses were done

X26) REB/IRB Approval and Ethical Considerations [recommended as subheading under "Methods"] (not a CONSORT item)

X26-i) Comment on ethics committee approval

subitem not at all important

1

2

3

4

5
essential
Does your paper address subitem X26-i?
Copy and paste relevant sections from the manuscript (include quotes in quotation marks "like this" to indicate direct quotes from your manuscript), or elaborate on this item by providing additional information not in the ms, or briefly explain why the item is not applicable/relevant for your study
Your answer

"Ethical
approval was applied for and received from the Deutsche Gesellschaft fürPsychologie (DGP; German Association for Psychology).It was conducted
in line with the APA ethical principles and the 1964 Helsinki Declaration and its later amendments or comparable ethical standards.A clinical trial registration at ClinicalTrials.gov was conducted for the

x26-ii) Outline informed consent procedures
Outline informed consent procedures e.g., if consent was obtained offline or online (how? Checkbox, etc.?), and what information was provided (see 4a-ii). See [6] for some items to be included in informed consent documents.
subitem not at all important
1
2
3
4
5
essential
Does your paper address subitem X26-ii?
Copy and paste relevant sections from the manuscript (include quotes in quotation marks "like this" to indicate direct quotes from your

manuscript), or elaborate on this item by providing additional information not in the ms, or briefly explain why the item is not applicable/relevant for your study

Your answer

"After
signing the consent form, each participant received a questionnaire and

X26-iii) Safety and security procedures

Safety and security procedures, incl. privacy considerations, and any steps taken to reduce the likelihood or detection of harm (e.g., education and training, availability of a hotline)

subitem not at all important

1

2

3

4

5

essential

Does your paper address subitem X26-iii?

Copy and paste relevant sections from the manuscript (include quotes in quotation marks "like this" to indicate direct quotes from your manuscript), or elaborate on this item by providing additional information not in the ms, or briefly explain why the item is not applicable/relevant for your study

Your answer

"The
researchers introduced themselves to participants and provided
information sheets, which contained both the aim and

RESULTS

13a) For each group, the numbers of participants who were randomly assigned, received intended treatment, and were analysed for the primary outcome

NPT: The number of care providers or centers performing the intervention in each group and the number of patients treated by each care provider in each center

Does your paper address CONSORT subitem 13a? *

Copy and paste relevant sections from the manuscript (include quotes in quotation marks "like this" to indicate direct quotes from your manuscript), or elaborate on this item by providing additional information not in the ms, or briefly explain why the item is not applicable/relevant for your study

Your answer

"...photo novel (N=64) and traditional brochure (N=62)." "..paper (N=66) and tablet formats (N=60)"

13b) For each group, losses and exclusions after randomisation, together with reasons

Does your paper address CONSORT subitem 13b? (NOTE: Preferably, this is shown in a CONSORT flow diagram) *

Copy and paste relevant sections from the manuscript (include quotes in quotation marks "like this" to indicate direct quotes from your manuscript), or elaborate on this item by providing additional information not in the ms, or briefly explain why the item is not

applicable/relevant for your study
Your answer

N/A, participants were randomized and trial

13b-i) Attrition diagram
Strongly recommended: An attrition diagram (e.g., proportion of participants still logging in or using the intervention/comparator in each group plotted over time, similar to a survival curve) or other figures or tables demonstrating usage/dose/engagement.
subitem not at all important
1
2
3
4
5
essential
Does your paper address subitem 13b-i?
Copy and paste relevant sections from the manuscript or cite the figure number if applicable (include quotes in quotation marks "like this" to indicate direct quotes from your manuscript), or elaborate on this item by providing additional information not in the ms, or briefly explain why the item is not applicable/relevant for your study
Your answer

There was no attrition issue so there is no attrition

14a) Dates defining the periods of recruitment and follow-up

Does your paper address CONSORT subitem 14a? *

Copy and paste relevant sections from the manuscript (include quotes in quotation marks "like this" to indicate direct quotes from your manuscript), or elaborate on this item by providing additional information not in the ms, or briefly explain why the item is not applicable/relevant for your study

Your answer

N/A, there was no recruitment and follow-up,

14a-i) Indicate if critical “secular events” fell into the study period

Indicate if critical “secular events” fell into the study period, e.g.,

significant changes in Internet resources available or “changes in computer hardware or Internet delivery resources”
subitem not at all important
1
2
3
4
5
essential
Does your paper address subitem 14a-i?
Copy and paste relevant sections from the manuscript (include quotes in quotation marks "like this" to indicate direct quotes from your manuscript), or elaborate on this item by providing additional information not in the ms, or briefly explain why the item is not applicable/relevant for your study
Your answer
No secular events fell into the study period
14b) Why the trial ended or was stopped (early)

Does your paper address CONSORT subitem 14b? *
Copy and paste relevant sections from the manuscript (include quotes in quotation marks "like this" to indicate direct quotes from your manuscript), or elaborate on this item by providing additional information not in the ms, or briefly explain why the item is not applicable/relevant for your study
Your answer

The trial was not stopped early

15) A table showing baseline demographic and clinical characteristics for each group

NPT: When applicable, a description of care providers (case volume, qualification, expertise, etc.) and centers (volume) in each group
Does your paper address CONSORT subitem 15? *
Copy and paste relevant sections from the manuscript (include quotes in quotation marks "like this" to indicate direct quotes from your manuscript), or elaborate on this item by providing additional information not in the ms, or briefly explain why the item is not applicable/relevant for your study
Your answer

"Both
 means and standard
deviations for each variable
of perceived health,
frequency of doctor visits,
and communication self-
efficacy (Ask,
[illegible]

15-i) Report demographics associated with digital divide issues
In ehealth trials it is particularly important to report demographics associated with digital divide issues, such as age, education, gender, social-economic status, computer/Internet/ehealth literacy of the participants, if known.
subitem not at all important
1
2
3
4
5
essential
Does your paper address subitem 15-i? *
Copy and paste relevant sections from the manuscript (include quotes in quotation marks "like this" to indicate direct quotes from your manuscript), or elaborate on this item by providing additional information not in the ms, or briefly explain why the item is not applicable/relevant for your study
Your answer

This is not addressed
explicitly, as the trial itself

16) For each group, number of participants (denominator) included in each analysis and whether the analysis was by original assigned groups

16-i) Report multiple “denominators” and provide definitions
Report multiple “denominators” and provide definitions: Report N’s (and effect sizes) “across a range of study participation [and use] thresholds” [1], e.g., N exposed, N consented, N used more than x times, N used more than y weeks, N participants “used” the intervention/comparator at specific pre-defined time points of interest (in absolute and relative numbers per group). Always clearly define “use” of the intervention.
subitem not at all important
1
2

3
4
5
essential
Does your paper address subitem 16-i? *
Copy and paste relevant sections from the manuscript (include quotes in quotation marks "like this" to indicate direct quotes from your manuscript), or elaborate on this item by providing additional information not in the ms, or briefly explain why the item is not applicable/relevant for your study
Your answer

N is reported for all reported results, but no multiple denominators were necessary

16-ii) Primary analysis should be intent-to-treat
Primary analysis should be intent-to-treat, secondary analyses could include comparing only “users”, with the appropriate caveats that this is no longer a randomized sample (see 18-i).
subitem not at all important
1
2
3
4
5
essential
Does your paper address subitem 16-ii?
Copy and paste relevant sections from the manuscript (include quotes in quotation marks "like this" to indicate direct quotes from your manuscript), or elaborate on this item by providing additional information not in the ms, or briefly explain why the item is not applicable/relevant for your study
Your answer

All of the analyses only compare information

17a) For each primary and secondary outcome, results for each group, and the estimated effect size and its precision (such as 95% confidence interval)

Does your paper address CONSORT subitem 17a? *

Copy and paste relevant sections from the manuscript (include quotes in quotation marks "like this" to indicate direct quotes from your manuscript), or elaborate on this item by providing additional information not in the ms, or briefly explain why the item is not applicable/relevant for your study

Your answer

"Although
 there was no statistically significant group difference among the
conditions in the evaluation variables($F(21,334)=1.34$, $P=.15$; Wilk's
[illegible]=0.79, partial [illegible]2=.075), the results show some significant differences
in some variables of the evaluation items based on certain conditions.
Namely, the variables of the evaluation item that accessed the understanding of the condition of brochure and format ($F(3,122)=2.91$

17a-i) Presentation of process outcomes such as metrics of use and intensity of use

In addition to primary/secondary (clinical) outcomes, the presentation of process outcomes such as metrics of use and intensity of use (dose, exposure) and their operational definitions is critical. This does not only refer to metrics of attrition (13-b) (often a binary variable), but also to more continuous exposure metrics such as “average session length”. These must be accompanied by a technical description how a metric like a “session” is defined (e.g., timeout after idle time) [1] (report under item 6a).

subitem not at all important

1

2

3

4

5

essential

Does your paper address subitem 17a-i?

Copy and paste relevant sections from the manuscript (include quotes in quotation marks "like this" to indicate direct quotes from your manuscript), or elaborate on this item by providing additional information not in the ms, or briefly explain why the item is not applicable/relevant for your study

Your answer

There were no doses or metrics

17b) For binary outcomes, presentation of both absolute and relative effect sizes is recommended

Does your paper address CONSORT subitem 17b? *

Copy and paste relevant sections from the manuscript (include quotes in quotation marks "like this" to indicate direct quotes from your manuscript), or elaborate on this item by providing additional information not in the ms, or briefly explain why the item is not applicable/relevant for your study

Your answer

No binary outcomes were assessed

18) Results of any other analyses performed, including subgroup analyses and adjusted analyses, distinguishing pre-specified from exploratory

Does your paper address CONSORT subitem 18? *

Copy and paste relevant sections from the manuscript (include quotes in quotation marks "like this" to indicate direct quotes from your manuscript), or elaborate on this item by providing additional information not in the ms, or briefly explain why the item is not

applicable/relevant for your study
Your answer
No adjusted analyses were performed
18-i) Subgroup analysis of comparing only users
A subgroup analysis of comparing only users is not uncommon in ehealth trials, but if done, it must be stressed that this is a self-selected sample and no longer an unbiased sample from a randomized trial (see 16-iii).
subitem not at all important
1
2
3
4
5
essential
Does your paper address subitem 18-i?
Copy and paste relevant sections from the manuscript (include quotes in quotation marks "like this" to indicate direct quotes from your manuscript), or elaborate on this item by providing additional information not in the ms, or briefly explain why the item is not applicable/relevant for your study
Your answer
N/A, all participants in each condition 'used' the material
19) All important harms or unintended effects in each group

(for specific guidance see CONSORT for harms)

Does your paper address CONSORT subitem 19? *

Copy and paste relevant sections from the manuscript (include quotes in quotation marks "like this" to indicate direct quotes from your manuscript), or elaborate on this item by providing additional information not in the ms, or briefly explain why the item is not applicable/relevant for your study

Your answer

There were none

19-i) Include privacy breaches, technical problems

Include privacy breaches, technical problems. This does not only include physical “harm” to participants, but also incidents such as perceived or real privacy breaches [1], technical problems, and other unexpected/unintended incidents. “Unintended effects” also includes unintended positive effects [2].
subitem not at all important
1
2
3
4
5
essential
Does your paper address subitem 19-i?
Copy and paste relevant sections from the manuscript (include quotes in quotation marks "like this" to indicate direct quotes from your manuscript), or elaborate on this item by providing additional information not in the ms, or briefly explain why the item is not applicable/relevant for your study
Your answer
There were none
19-ii) Include qualitative feedback from participants or observations from staff/researchers
Include qualitative feedback from participants or observations from staff/researchers, if available, on strengths and shortcomings of the application, especially if they point to unintended/unexpected effects or uses. This includes (if available) reasons for why people did or did not use the application as intended by the developers.
subitem not at all important
1
2
3
4
5
essential
Does your paper address subitem 19-ii?
Copy and paste relevant sections from the manuscript (include quotes in

quotation marks "like this" to indicate direct quotes from your manuscript), or elaborate on this item by providing additional information not in the ms, or briefly explain why the item is not applicable/relevant for your study

Your answer

This was not included in the study

DISCUSSION

22) Interpretation consistent with results, balancing benefits and harms, and considering other relevant evidence

NPT: In addition, take into account the choice of the comparator, lack of or partial blinding, and unequal expertise of care providers or centers in each group
22-i) Restate study questions and summarize the answers suggested by the data, starting with primary outcomes and process outcomes (use)
Restate study questions and summarize the answers suggested by the data, starting with primary outcomes and process outcomes (use).
subitem not at all important
1
2
3

4
5
essential
Does your paper address subitem 22-i? *
Copy and paste relevant sections from the manuscript (include quotes in quotation marks "like this" to indicate direct quotes from your manuscript), or elaborate on this item by providing additional information not in the ms, or briefly explain why the item is not applicable/relevant for your study
Your answer

"An
 evaluation of different forms of patient interventions with 126 older
adults in Germany revealed that the photo novel intervention was more positively evaluatedin comparison to traditional brochures: Participants
 claimed that a photo novel is easier to understand, more enjoyable and
more informative than a traditional brochure. Furthermore, the current study discovered that older adults preferred paper format to tablet
format, due to paper beingless monotonous, less boring and not too long,
 with the length of the contents being judged as appropriate. Overall,
the current study participants preferred the paper format to the electronic device (in a tablet format). In addition, this study examined
 four conditions and discovered that a photo novel on a tablet
(Condition 4) was easier to understand in comparison to a traditional
[illegible]

brochure on a tablet
(Condition 2). The condition of a traditional
brochure on a tablet appeared to be less effective since it was
considered to be more monotonous compared to the paper formats of both
the traditional brochure and photo novel (Condition 1 and 3). Along this
line, the participants rated Condition 2 (traditional brochure on a
tablet) as more boring and too long to read, especially in comparison to
Condition 1 (traditional brochure on paper). In

22-ii) Highlight unanswered new questions, suggest future research
Highlight unanswered new questions, suggest future research.
subitem not at all important
1
2
3
4
5
essential
Does your paper address subitem 22-ii?
Copy and paste relevant sections from the manuscript (include quotes in quotation marks "like this" to indicate direct quotes from your manuscript), or elaborate on this item by providing additional information not in the ms, or briefly explain why the item is not applicable/relevant for your study
Your answer

"Therefore,
the use of a photo novel in health interventions, and the use of
technology, are worth investigating further in the future." "In future
studies, beside larger sample sizes, longer follow-up [illegible]

20) Trial limitations, addressing sources of potential bias, imprecision, and, if relevant, multiplicity of analyses

20-i) Typical limitations in ehealth trials

Typical limitations in ehealth trials: Participants in ehealth trials are

rarely blinded. Ehealth trials often look at a multiplicity of outcomes, increasing risk for a Type I error. Discuss biases due to non-use of the intervention/usability issues, biases through informed consent procedures, unexpected events.
subitem not at all important
1
2
3
4
5
essential
Does your paper address subitem 20-i? *
Copy and paste relevant sections from the manuscript (include quotes in quotation marks "like this" to indicate direct quotes from your manuscript), or elaborate on this item by providing additional information not in the ms, or briefly explain why the item is not applicable/relevant for your study
Your answer
"Despite
the study having a
reasonable sample size, more
participants for the
different conditions would
have been more favourable to
obtain more
power to detect statistical
significance [28]. In future
studies, beside
larger sample sizes, longer
21) Generalisability (external validity, applicability) of the trial findings

NPT: External validity of the trial findings according to the intervention, comparators, patients, and care providers or centers involved in the trial

21-i) Generalizability to other populations

Generalizability to other populations: In particular, discuss generalizability to a general Internet population, outside of a RCT setting, and general patient population, including applicability of the study results for other organizations

subitem not at all important

1

2

3
4
5
essential
Does your paper address subitem 21-i?
Copy and paste relevant sections from the manuscript (include quotes in quotation marks "like this" to indicate direct quotes from your manuscript), or elaborate on this item by providing additional information not in the ms, or briefly explain why the item is not applicable/relevant for your study
Your answer

"These findings might be specific for the current generation of older adults in Germany. "

21-ii) Discuss if there were elements in the RCT that would be different in a routine application setting
Discuss if there were elements in the RCT that would be different in a routine application setting (e.g., prompts/reminders, more human involvement, training sessions or other co-interventions) and what impact the omission of these elements could have on use, adoption, or outcomes if the intervention is applied outside of a RCT setting.
subitem not at all important
1
2
3
4
5
essential
Does your paper address subitem 21-ii?
Copy and paste relevant sections from the manuscript (include quotes in quotation marks "like this" to indicate direct quotes from your manuscript), or elaborate on this item by providing additional information not in the ms, or briefly explain why the item is not applicable/relevant for your study
Your answer

N/A, this would not be different in a routine

OTHER INFORMATION

23) Registration number and name of trial registry

Does your paper address CONSORT subitem 23? *

Copy and paste relevant sections from the manuscript (include quotes in quotation marks "like this" to indicate direct quotes from your manuscript), or elaborate on this item by providing additional information not in the ms, or briefly explain why the item is not applicable/relevant for your study

Your answer

"A clinical trial registration at ClinicalTrials.gov was conducted for the present study (NCT02502292)...."

24) Where the full trial protocol can be accessed, if available

Does your paper address CONSORT subitem 24? *
Cite a Multimedia Appendix, other reference, or copy and paste relevant sections from the manuscript (include quotes in quotation marks "like this" to indicate direct quotes from your manuscript), or elaborate on this item by providing additional information not in the ms, or briefly explain why the item is not applicable/relevant for your study
Your answer

"A
clinical trial registration at ClinicalTrials.gov was conducted for the
present study (NCT02502292), in which the description of this study with its objectives, designs, methodologies, and the [illegible]

25) Sources of funding and other support (such as supply of drugs), role of funders

Does your paper address CONSORT subitem 25? *

Copy and paste relevant sections from the manuscript (include quotes in

quotation marks "like this" to indicate direct quotes from your manuscript), or elaborate on this item by providing additional information not in the ms, or briefly explain why the item is not applicable/relevant for your study

Your answer

"We
thank the European Union for their grant which made it possible to
conduct this research.
Funding stems from the European Union's Seventh Framework Programme (FP7/2007-2013) under grant agreement nº305831.
Parts of this research was funded by the Wilhelm-Stiftung für Rehabilitationsforschung within the Donors'Association for the Promotion
of Humanities and Sciences

X27) Conflicts of Interest (not a CONSORT item)

X27-i) State the relation of the study team towards the system being evaluated

In addition to the usual declaration of interests (financial or otherwise), also state the relation of the study team towards the system being evaluated, i.e., state if the authors/evaluators are distinct from or identical with the developers/sponsors of the intervention.

subitem not at all important

1
2
3
4

5

essential

Does your paper address subitem X27-i?

Copy and paste relevant sections from the manuscript (include quotes in quotation marks "like this" to indicate direct quotes from your manuscript), or elaborate on this item by providing additional information not in the ms, or briefly explain why the item is not applicable/relevant for your study

Your answer

"The authors declare that they have no competing

About the CONSORT EHEALTH checklist

As a result of using this checklist, did you make changes in your manuscript? *
yes, major changes
yes, minor changes
no
What were the most important changes you made as a result of using this checklist?
Your answer
Including relevant words in the title, some specifics of
How much time did you spend on going through the checklist

INCLUDING making changes in your manuscript *
Your answer

2 hours

As a result of using this checklist, do you think your manuscript has improved? *
yes
no
Other:

Slightly

Would you like to become involved in the CONSORT EHEALTH group?
This would involve for example becoming involved in participating in a workshop and writing an "Explanation and Elaboration" document
yes
no
Other:

Any other comments or questions on CONSORT EHEALTH
Your answer

STOP - Save this form as PDF before you click submit

To generate a record that you filled in this form, we recommend to generate a PDF of this page (on a Mac, simply select "print" and then select "print as PDF") before you submit it.

When you submit your (revised) paper to JMIR, please upload the PDF as supplementary file.

Don't worry if some text in the textboxes is cut off, as we still have the complete information in our database. Thank you!

Final step: Click submit !

Click submit so we have your answers in our database!

Submit

Never submit passwords through Google Forms.

This content is neither created nor endorsed by Google. Report Abuse - Terms of Service - Additional Terms

Google Forms
